# The influence of astrocytes on the width of orientation hypercolumns in visual cortex: A computational perspective

**DOI:** 10.1371/journal.pcbi.1005785

**Published:** 2017-10-27

**Authors:** Ryan T. Philips, Mriganka Sur, V. Srinivasa Chakravarthy

**Affiliations:** 1 Computational Neuroscience Laboratory, Department of Biotechnology, Bhupat and Jyoti Mehta School of Biosciences, Indian Institute of Technology Madras, Chennai, Tamil Nadu, India; 2 Picower Institute for Learning and Memory, Department of Brain and Cognitive Sciences, Massachusetts Institute of Technology, Cambridge, Massachusetts, United States of America; University of Pittsburgh, UNITED STATES

## Abstract

Orientation preference maps (OPMs) are present in carnivores (such as cats and ferrets) and primates but are absent in rodents. In this study we investigate the possible link between astrocyte arbors and presence of OPMs. We simulate the development of orientation maps with varying hypercolumn widths using a variant of the Laterally Interconnected Synergetically Self-Organizing Map (LISSOM) model, the Gain Control Adaptive Laterally connected (GCAL) model, with an additional layer simulating astrocytic activation. The synaptic activity of V1 neurons is given as input to the astrocyte layer. The activity of this astrocyte layer is now used to modulate bidirectional plasticity of lateral excitatory connections in the V1 layer. By simply varying the radius of the astrocytes, the extent of lateral excitatory neuronal connections can be manipulated. An increase in the radius of lateral excitatory connections subsequently increases the size of a single hypercolumn in the OPM. When these lateral excitatory connections become small enough the OPM disappears and a salt-and-pepper organization emerges.

## Author summary

Columns of neurons in the primary visual cortex (V1) are known to be tuned to visual stimuli containing edges of a particular orientation. The arrangement of these cortical columns varies across species. In many species such as in ferrets, cats, and monkeys a topology preserving map is observed, wherein similarly tuned columns are observed in close proximity to each other, resulting in the formation of Orientation Preference Maps (OPMs). The width of the hypercolumns, the fundamental unit of an OPM, also varies across species. However, such an arrangement is not observed in rodents, wherein a more random arrangement of these cortical columns is reported. We explore the role of astrocytes in the arrangement of these cortical columns using a self-organizing computational model. Invoking evidence that astrocytes could influence bidirectional plasticity among effective lateral excitatory connections in V1, we introduce a mechanism by which astrocytic inputs can influence map formation in the neuronal network. In the resulting model-generated OPMs the radius of the hypercolumns is found to be correlated with that of astrocytic arbors, a feature that finds support in experimental studies.

## Introduction

The cortex is the outermost layer of cerebral tissue, composed of neuronal cell bodies and protoplasmic astroytes. The neurons in the cortex are arranged in columns, and the neurons in each column usually respond to similar features. In the macaque these columns, known as microcolumns or minincolumns have a density of 1270 minicolumns per *mm*^2^, with each minicolumn having around 142 pyramidal cell bodies [1]. Now the 3-d volume of cortical tissue could be locally approximated as a 2-d sheet of nodes, with a single node representative of all the neurons within a particular column. With this approximation it becomes possible to describe a 2-d map in the neuronal space with each node responding to a particular feature in the stimulus space.

A number of such stimulus modality-specific feature maps are topographic in nature, meaning that features that are similar in the stimulus space are mapped onto neighboring locations in the cortical space. A few examples include the tactile map in the primary somatosensory cortex [2], the whisker map in the barrel cortex [3], and the orientation, direction and retinotopic maps in the primary visual cortex [4]. Understanding the mapping function allows prediction of what features a particular neuron will respond to. A model which simulates the development of such maps, would aid in understanding which factors contribute to the development of such features maps. These factors could include internal factors such as the connectivity between the nodes, or the available area of the cortex onto which the features are to be mapped. Similarly features of the stimuli used for training the model themselves act as external factors.

Self-organizing maps (SOMs) have been used extensively to simulate the development of cortical maps [5–12]. A SOM has two constraints: coverage and continuity. Optimal coverage implies all input stimuli are mapped evenly on to the output space. Continuity implies that neighboring neurons in the output space respond to similar stimuli. The SOM uses local learning rules in order to optimize coverage and continuity. A biologically realistic variant of SOM, namely the Gain Controlled Adaptive Lateral (GCAL), has been used to investigate the factors involved in the development of a number of feature maps in the primary visual cortex (V1) [13]. The GCAL model consists of sheets of neurons. Each neuron in each layer could have 3 kinds of connections, each of which is trained using a normalized Hebbian learning rule:

- Afferent Excitatory: These are connections from nodes in another sheet which are excitatory in nature.
- Lateral Excitatory: These are connections from nodes in the same sheet which are excitatory in nature. These are short ranged connections.
- Lateral Inhibitory: These are connections from nodes in the same sheet which are inhibitory in nature. These are longer ranged connections.

A common feature in most SOMs is the presence of a mechanism by which neighboring neurons respond to similar features whereas those further away respond to dissimilar ones. The GCAL model achieves this by having short range excitatory connections, and longer range inhibitory connections. However in V1 inhibitory connections are local (short range) and may dominate responses [14], whereas the long range connections are excitatory. The effective long range inhibition is achieved by excitatory neurons synapsing onto inhibitory neurons which in turn synapse onto other neurons in its vicinity. For high contrast stimuli, it is known that the long range connections are in effect (multi-synapse) inhibitory in nature [15]. From a computational perspective, the radius of the effectively short range excitatory connections is important in determining the size of the orientation hypercolumn [6, 16]. In the absence of any excitatory connections, with Hebbian trained afferent connections and anti-Hebbian trained lateral connections, a sparse representation yielding independent components of the training set is realized [17]. This implies that an OPM will give way to a salt-and-pepper organization, without a smooth shift in orientation preference among neighboring neurons, in the absence of lateral excitatory connections. OPMs are present in carnivores (such as cats and ferrets) and primates but absent in rodents [18]. The term ‘salt-and-pepper’ was originally used to describe the maps seen in rodents, since the orientation preference of neighboring neuronal columns appeared to be uncorrelated and resembled a random pattern. However, recent experimental evidence suggests that the map is pseudo-random and exhibits some local similarities in orientation preference [19].

We hypothesize a possible link between astrocytic arbors and presence of OPMs and try to show that larger astrocytic arbors are more conducive to the generation of OPMs. We investigate the above hypothesis using computational modeling. We propose a GCAL model having 2 V1 layers: one representative of neurons, whereas the other of astrocytes. The synaptic activity of V1 neurons is given as input to an astrocyte layer. The activity of the astrocyte layer is now used to modulate bidirectional plasticity of lateral excitatory connections in the V1 layer. By simply varying the radius of astrocytes, the effective extent of lateral excitatory neuronal connections can be manipulated. An increase in the effective radius of lateral excitatory connections subsequently increases the size of a single hypercolumn in the OPM. When these effective lateral excitatory connections become small enough the OPM disappears and a salt-and-pepper organization emerges.

Hubel and Wiesel proposed that the emergence of orientation preference in principal (layer 4) neurons in the primary visual cortex is primarily due to the spatial arrangement of LGN afferent connections [20], though the effect of recurrent connections is now also clear. This contribution of afferent feed-forward connections is also emphasized by Paik and Ringach, who attribute the development of orientation preference maps across species to the Moire interference patterns created due to the spatial arrangement of Retinal Ganglion Cells (RGCs) [21]. While the contribution of feed forward connections to map formation is undeniable, as verified by a number of experiments, the contribution of recurrent lateral connections between cortical columns is also prominent. At the level of columns, rather than at the level of single neuron, it is known that for high contrast inputs, due to the recruitment of local inhibitory inter-neurons, long range lateral connections are predominantly inhibitory in nature [15, 22, 23]. This configuration of lateral connections is essential for map formation [7]. What shapes the lateral circuitry in cortical networks? Are there mechanisms which could ensure that short range connections are excitatory, whereas as the long range connections are in effect (considering the contribution of interneurons) predominantly inhibitory?

We hypothesize that protoplasmic astrocytes could play a key role in this regard. Although there are a number of mechanisms by which astrocytes and neurons communicate with each other [24–27], not all these mechanisms contribute to long term plasticity, crucial for the development of cortical maps. It must however be noted that there are a number of ways in which astrocytes could influence long term plasticity. These mechanisms could be summarized as follows:

- Astrocyte calcium levels could regulate response levels and selectivity of local neurons by altering both excitation and inhibition in local circuits [28].
- Protoplasmic astrocytes are reported to have increased intracellular calcium levels in response to synaptic glutamate via transporters/ metabotropic receptors [29].
- Increased calcium levels in astrocytes are believed to trigger the release of gliotransmitters such as glutamate and D-Serine; however, there is still an ongoing debate regarding the multitude of factors required for the release of gliotransmitters (for review see [30]).
- There is evidence that these gliotransmitters could contribute to bidirectional plasticity (for review see [30]). In particular D-Serine acts as a co-agonist for post synaptic NMDA receptors and is known to affect meta-plasticity [31].
- Gliotransmitters could also activate presynaptic receptors leading to an increase in synaptic release probability [32].
- Similarly at the postsynaptic neuron, gliotrasmitters could influence the number or strength of the receptor channels [33].

In each of these mechanisms the effective synaptic strength is influenced by astrocytic activity. NMDA-dependent LTP/LTD is known to be a function of the postsynaptic calcium influx [34]. The postsynaptic calcium influx is likely dependent on astrocytic activity as well. The astrocytic influence could be abstracted using a plasticity or learning rule (such as a BCM curve), where the threshold controlling LTP vs. LTD is dependent on the astrocytic activity. The lateral excitatory connections in the modified GCAL model are modeled in such a manner.

## Methods

### Gain control, adaptation laterally connected model

The Gain Control, Adaptation, Laterally Connected (GCAL) model, has been used to develop stable and robust orientation maps [35]. This model builds on the LISSOM model and has, as the name suggests, a mechanism which ensures gain control of input activations and homeostatic adaptation of weights. The model has 3 layers: a photo-receptive input layer, an ON/OFF LGN layer and a V1 layer.

The activity of the ON/OFF LGN layer is given as *L* for a node *i*, *j* in the layer.

$$\begin{array}{c} L_{i,j}\left(t+1\right)=f(\frac{\gamma_{o}\sum_{a,b}x_{a,b}\left(t\right)C_{ij,ab}}{k+\gamma_{s}\sum_{a,b}L_{i,j}\left(t\right)C_{ij,ab}^{s}}) \end{array}$$ (1)

where (*a*, *b*) denotes a neuron in the receptive field of the (*i*, *j*)^*th*^ neuron in the output layer, with input given as *x*_*ab*_; *C*_*ij*, *ab*_ represents the weight from the (*a*, *b*)^*th*^ neuron to the (*i*, *j*)^*th*^ neuron. A constant multiplier to the overall strength is given by *γ*_*o*_; *γ*_*s*_ represents the gain-control. The weights *C*_*ij*, *ab*_ are defined as a difference of Gaussians.

$$\begin{array}{c} C_{ij,ab}=\frac{1}{Z_{c}}exp(-\frac{{\left(a-i\right)}^{2}+{\left(b-j\right)}^{2}}{2\sigma_{c}^{2}})-\frac{1}{Z_{s}}exp(-\frac{{\left(a-i\right)}^{2}+{\left(b-j\right)}^{2}}{2\sigma_{s}^{2}}) \end{array}$$ (2)

where *Z*_*c*_, and *Z*_*s*_ denote the normalization factors, *σ*_*c*_, and *σ*_*s*_ regulate the width of the gaussians.

The term $C_{ij,ab}^{s}$ denotes the lateral inhibition received from other ON/OFF units.

$$\begin{array}{c} C_{ij,ab}^{s}=\frac{1}{Z_{s}}exp(-\frac{{\left(a-i\right)}^{2}+{\left(b-j\right)}^{2}}{2\sigma_{c}^{2}}) \end{array}$$ (3)

The firing rate of a V1 neuron is dependent on only 3 kinds of inputs, namely: afferent inputs from the LGN (*L*_*ab*_(*t* − 1)), lateral effectively excitatory inputs, and lateral effectively inhibitory inputs. Thus the firing rate (*y*_*ij*_(*t*)) is given as:

$$\begin{array}{c} y_{ij}\left(t\right)=f(p\sum_{a,b}A_{ij,ab}L_{ab}\left(t-1\right)+q\sum_{k,l}E_{ij,kl}y_{kl}\left(t-1\right)-r\sum_{k,l}I_{ij,kl}y_{kl}\left(t-1\right)) \end{array}$$ (4)

where *p*, *q*, *r* are scaling factors; *A*_*ij*, *ab*_ is the afferent weight from neuron (*a*, *b*) to neuron (*i*, *j*); *E*_*ij*, *kl*_ is the lateral excitatory weight from neuron (*k*, *l*) to neuron (*i*, *j*) and similarly *I*_*ij*, *kl*_ is the lateral inhibitory weight from neuron (*k*, *l*) to neuron (*i*, *j*). The function *f* is a half wave rectifier in order to ensure that the activations are positive with a variable threshold point given as *ρ*. The activations *y*_*ij*_(*t*) are allowed to adapt in a homeostatic fashion. The output activity *y*_*ij*_ and the threshold *ρ* are adapted as follows:

$$\begin{array}{c} {\bar{y}}_{ij}\left(t\right)=\left(1-\beta \right)y_{ij}\left(t\right)+\beta {\bar{y}}_{ij}\left(t-1\right) \end{array}$$ (5)

$$\begin{array}{c} \rho \left(t\right)=\rho \left(t-1\right)+\lambda {\bar{y}}_{ij}\left(t\right)-\mu \end{array}$$ (6)

where *β* is the smoothing parameter and λ is the homeostatic learning rate; ${\bar{y}}_{ij}\left(t\right)$ is initialized to the average V1 activity (*μ*).

In order to model astrocytic activation we simulate an additional layer whose input is the synaptic activity (*gs*) present at each node of the V1 layer. Thus the activation of a single node in this astrocyte layer is given by *S*_*ij*_.

$$\begin{array}{c} gs_{ij}\left(t\right)=p\sum_{a,b}A_{ij,ab}L_{ab}\left(t-1\right)+q\sum_{k,l}E_{ij,kl}y_{kl}\left(t-1\right)-r\sum_{k,l}I_{ij,kl}y_{kl}\left(t-1\right) \end{array}$$ (7)

$$\begin{array}{c} S_{ij}\left(t\right)=\sum_{i,j\in R_{astro}}gs_{ij}\left(t-1\right) \end{array}$$ (8)

where the radius of the astrocyte is given as *R*_*Astro*_. There is some debate regarding the precise nature of GABA induced calcium oscillations in the astrocyte and the subsequent gliotranmission [36]. Hence we run an additional simulation which does not consider the effect of GABA induced gliotransmitters. Now the activation of a single node in the astrocyte layer is given by *S*_*ij*_.

$$\begin{array}{c} gs_{ij}\left(t\right)=p\sum_{a,b}A_{ij,ab}L_{ab}\left(t-1\right)+q\sum_{k,l}E_{ij,kl}y_{kl}\left(t-1\right) \end{array}$$ (9)

$$\begin{array}{c} S_{ij}\left(t\right)=\sum_{i,j\in R_{astro}}gs_{ij}\left(t-1\right) \end{array}$$ (10)

The lateral inhibitory and afferent weights are trained using the same normalized Hebbian rule given by:

$$\begin{array}{c} w_{ij,mn}\left(t+1\right)=\frac{w_{ij,mn}\left(t\right)+\eta y_{ij}\left(t\right)P_{mn}\left(t\right)}{\sum_{mn}\left(w_{ij,mn}\left(t\right)+\eta y_{ij}\left(t\right)P_{mn}\left(t\right)\right)} \end{array}$$ (11)

where *P*_*mn*_ is the generalized notation for the pre-synaptic activity originating from the neuron (*m*, *n*); *η* is the learning rate. These learning rates can be different for each of the connections: *η*_*A*_, *η*_*E*_ and *η*_*I*_ are the learning rates for the afferent, excitatory and inhibitory connections respectively.

However the lateral excitatory connections adapt using a variant of the BCM rule with a threshold function *θ* being a function of the astrocytic activation at the corresponding node. It has been previously proposed that astrocytes introduce metaplasticity by shifting the BCM curve [31].

$$\begin{array}{c} E_{ij,kl}\left(t+1\right)=E_{ij,kl}\left(t\right)+\eta_{E}y_{ij}\left(t\right)\left(y_{ij}\left(t\right)-\theta_{ij}\right)y_{kl}\left(t\right) \end{array}$$ (12)

$$\begin{array}{c} \theta_{ij}=\left(1-S_{ij}\right) \end{array}$$ (13)

**Fig 1:**
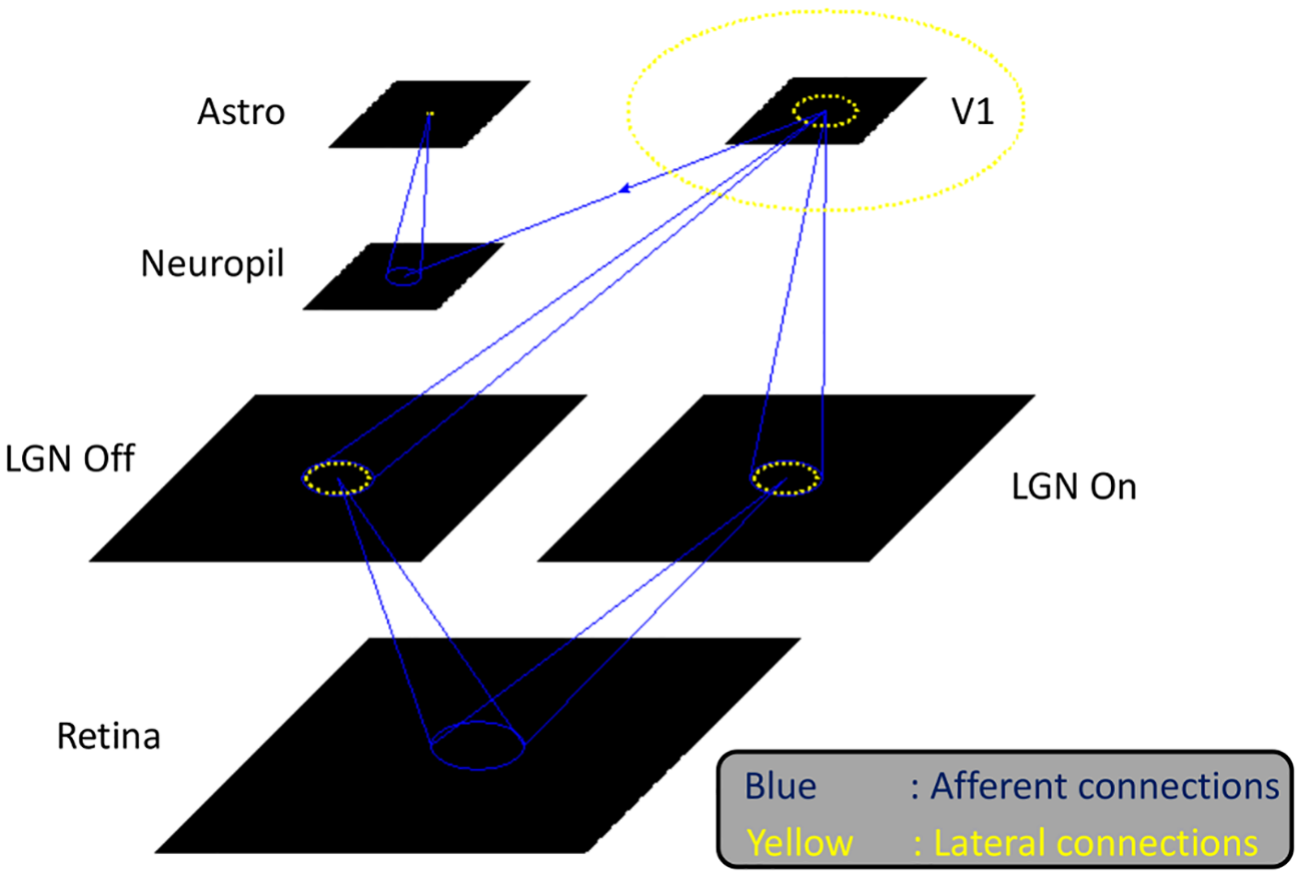
The GCAL model architecture used to simulate the effect of astrocytic feedback and the orientation hypercolumn width. The activity at the photoreceptor input layer is fed to the LGN ON/OFF layer which is simulated using a difference of Gaussians. The LGN activity then projects onto the V1 layer which has 3 kinds of connections: afferent, lateral excitatory and lateral inhibitory. The synaptic variable at the V1 layer, i.e. the activity of V1 neurons before passing it to the transfer function is assumed to form the neuropil layer. The activity at the neuropil layer is given to the astrocyte layer which could also have lateral excitatory connections which represent the gap junctions.

Astrocytes communicate with each other via gap junctions; however only distal branches are connected, resulting in astrocytic microdomains with less than 10% overlap [37]. The gap junctions could be modeled using Gaussian random lateral excitatory connections to the 8 nearest neighboring nodes. A schematic of the model is shown in Fig 1

The parameters used for the GCAL model are a superset of those used in the standard LISSOM model. The complete list of parameters are given in Table 1. The simulations are performed using the Topographica simulator [38].

**Table 1:** Variables of the GCAL model for the simulation of the orientation map.

Parameter	Value	Description
*p*	1.5	Afferent Strength
*q*	2.1	Lateral Excitatory Strength
*r*	1.4	Lateral Inhibitory Strength
*η*_*A*_	0.1	Afferent Learning Rate
*η*_*E*_	0.3	Excitatory Learning Rate
*η*_*I*_	0.3	Inhibitory Learning Rate
*rad*_*A*_	0.27	Afferent Radius
*rad*_*I*_	0.22	Inhibitory Radius
*R*_*Astro*_	-	Astrocytic Radius
*x*	-	Retinal neuronal activation
*L*	-	LGN neuronal activation
*y*	-	V1 neuronal activation
*gs*	-	V1 synaptic
*S*	-	V1 astrocytic activation
*A*	-	LGN to V1 weights
*E*	-	V1 to V1 excitatory weights
*I*	-	V1 to V1 inhibitory weights

### Analysis

#### Hypercolumn width

In order to estimate the width of the hypercolumn we use a formulation prescribed by [39]. We first compute the difference map I’(x)(0° -90°, 45° -45°) by subtracting the output layer responses for the corresponding orientations *y*_*ij*_.

$$\begin{array}{c} I\left(x\right)=I^{\prime }\left(x\right)-J\left(X\right) \end{array}$$ (14)

where J(x) is a smoothed version of I’(x)

$$\begin{array}{c} J\left(x\right)=\frac{1}{W(x)}F^{-1}S\left(k\right)I^{\prime }\left(k\right) \end{array}$$ (15)

where $F^{-1}$ denotes the inverse Fourier transform and *S* is the Fermi function given as:

$$\begin{array}{c} S\left(k\right)=\frac{1}{1+e^{(k_{hp}-\left|k|\right)/\beta_{hp}}} \end{array}$$ (16)

where *k*_*hp*_ is the high frequency cutoff and *β*_*hp*_ denotes the steepness. The normalizing function W(x) is given as:

$$\begin{array}{c} W\left(x\right)=\int S\left(x^{\prime }-x\right)d^{2}x^{\prime } \end{array}$$ (17)

Now the power spectrum of the filtered difference map is computed.

$$\begin{array}{c} P\left(k\right)={\left|I\left(k\right)\right|}^{2} \end{array}$$ (18)

On averaging over the angle

$$\begin{array}{c} P(k)=\int P(k,\theta )d\theta \end{array}$$ (19)

The dominant frequency *ζ* of the spectrum *P* is used to determine the hypercolumnar spacing.

In order to estimate the dominant frequency, the power spectrum *P* is fit with the function

$$\begin{array}{c} f\left(k\right)=a_{0}\exp(-\frac{{\left(k-a_{1}\right)}^{2}}{2a_{2}^{2}})+a_{3}+a_{4}k+a_{5}k^{2} \end{array}$$ (20)

where *a*_*i*_ are fitting parameters. The peak position is obtained by approximating *ζ* = *a*_1_.

#### Pinwheel identification

In order to estimate the pinwheel density of a given orientation map, first the location of each pinwheel needs to be identified and their total number estimated. The polar representation of orientation preference of each node is first computed. The intersection of the real and imaginary components of the polar representation of the zero contours give the pinwheel centers [39].

#### Stability index

In order to demonstrate that the maps developed remain roughly stable over iterations, the similarity between the developing maps is estimated using a correlation metric. This ‘orientation similarity index’ as utilized by [35] varies from 0.0 to 1.0, with 1.0 denoting perfect correlation. The stability index (SI) is given as:

$$\begin{array}{c} SI=1-\frac{4}{n\pi }\sum_{i}\left|\left(F_{i}-O_{i}\right)mod\left(\pi /2\right)\right| \end{array}$$ (21)

where *i* denotes the index of the pixels in the orientation preference maps consisting of *n* pixels; *F*, *O* denote the orientation preference maps at different time points in the development.

## Results

### Correlation between the astrocytic radius and the width of the hypercolumn

We vary the astrocytic radius and observe the changes in the orientation map developed. The experimentally reported astrocytic radii are estimated using the Glial fibrillary acidic protein (GFAP) as the astrocytic marker. However, the GFAP marked region accounts for only 15% of the actual astrocytic volume. Hence we scale the astrocytic radii by a factor of 2 in the simulations. The model is trained for 10000 iterations. The training regime consists of elongated 2-dimensional Gaussians with centers and orientations drawn from a uniform random distribution.

**Fig 2:**
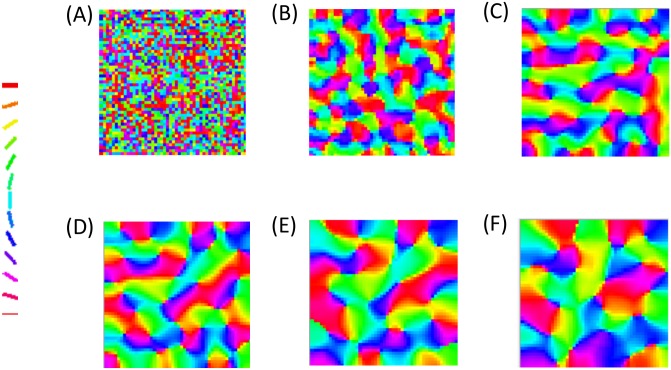
The astrocyte orientation maps developed on varying the radius of astrocytic influence *R*_*astro*_. The astrocytic radii used are (A) 0.015 mm, (B) 0.0375 mm, (C) 0.075 mm, (D) 0.1125 mm, (E) 0.15 mm, (F) 0.1875 mm.

**Fig 3:**
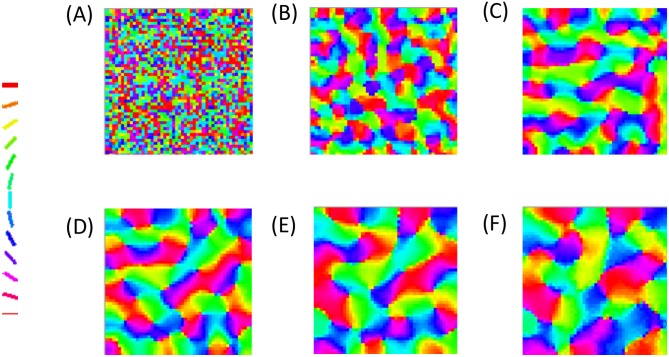
The neuronal (V1) orientation maps developed on varying the radius of astrocytic influence *R*_*astro*_. The astrocytic radii used are (A) 0.015 mm, (B) 0.0375 mm, (C) 0.075 mm, (D) 0.1125 mm, (E) 0.15 mm, (F) 0.1875 mm.

**Fig 4:**
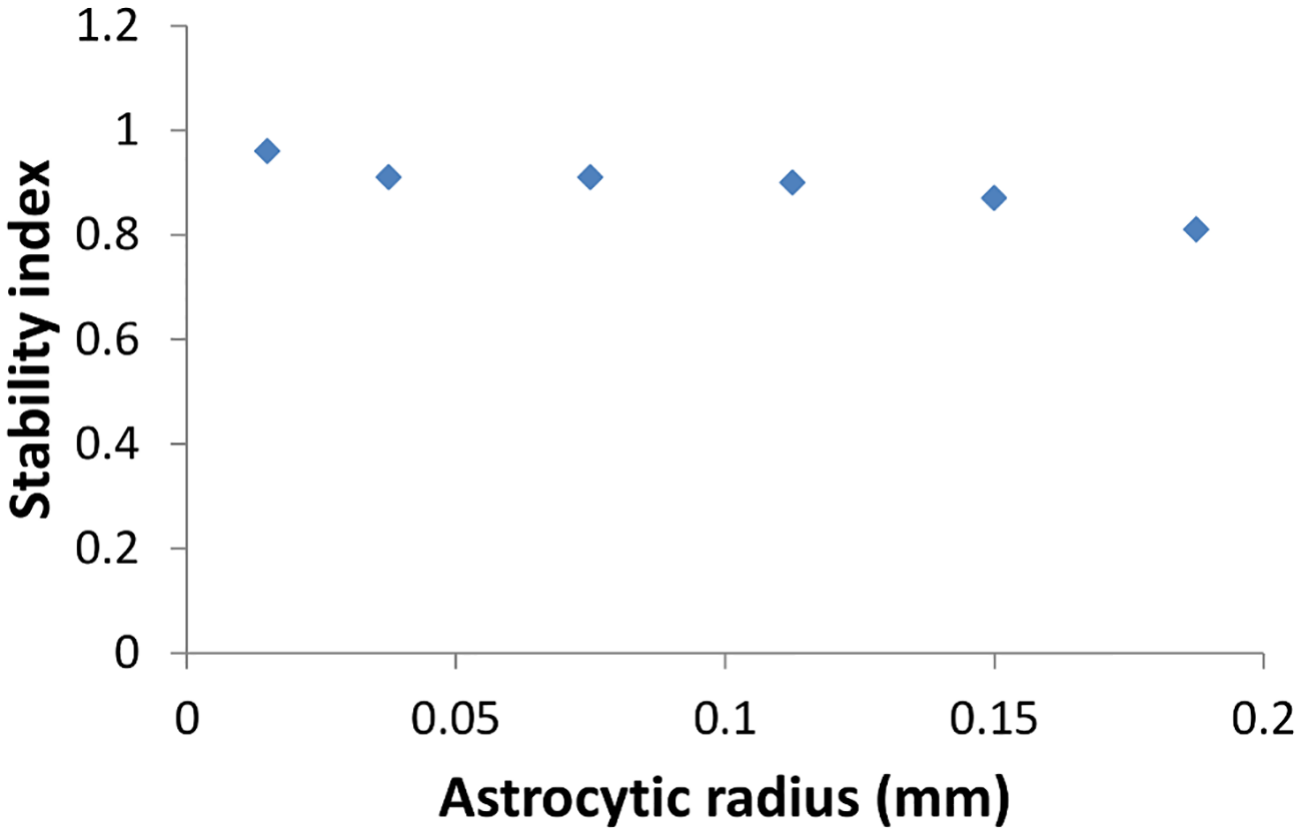
The correlation between the neuronal and astrocytic orientation maps as quantified by the stability index on varying *R*_*Astro*_.

The astrocytic radius is varied and the corresponding orientation maps developed are studied (Figs 2 and 3). It is observed that on reducing the astrocytic radius, the periodicity of the map increases and the width of a single hypercolumn decreases. Thus in a given area of cortical tissue 3 x 3 mm, the number of orientation hypercolumns would increase as we reduce the astrocytic radius. The neuronal and astrocyte maps developed have similar orientation preferences which could be quantified by their stability index. The stability index between the astrocytic and orientation maps is shown in Fig 4. These results demonstrate that the astrocyte radius has a profound effect on OPM formation.

**Fig 5:**
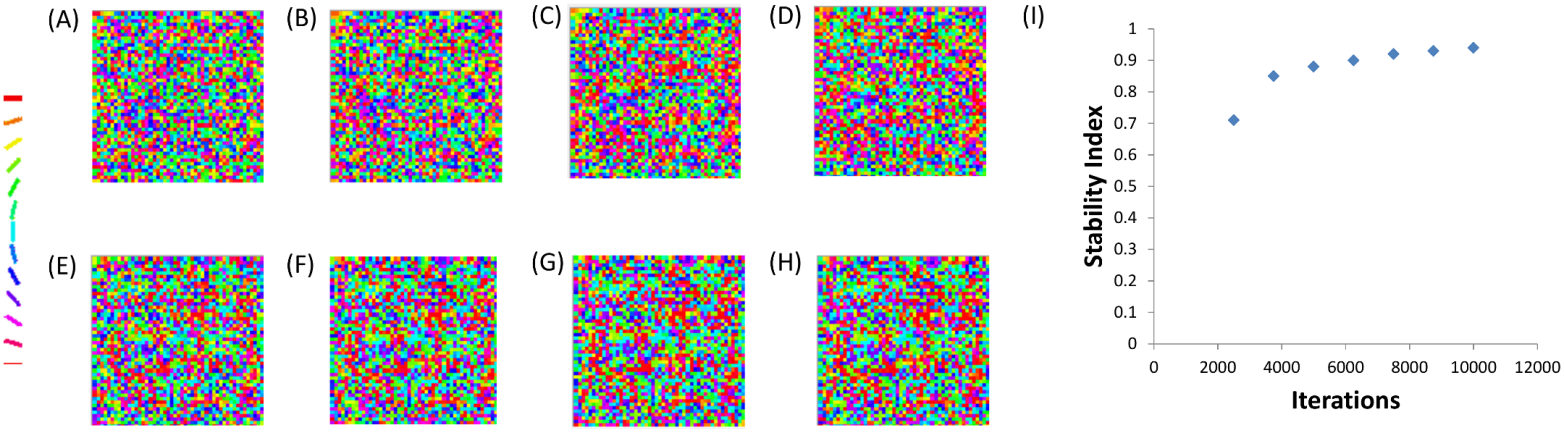
Development of the neuronal (V1) orientation preference map with the astrocyte radius *R*_*astro*_ set to 0.015 mm. The V1 orientation preference map when probed at A: 250, B: 500, C: 750, D: 1000, E: 2500, F: 5000, G: 7500, H: 10000 iterations respectively. I: The stability of the V1 orientation map developed across iterations.

**Fig 6:**
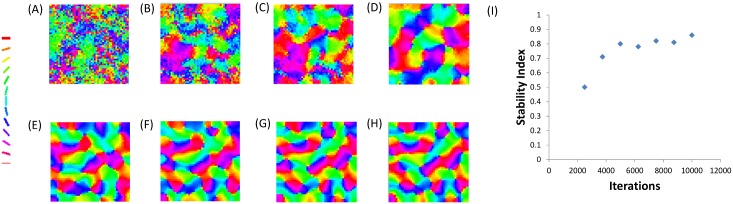
Development of the neuronal (V1) orientation preference map with the astrocyte radius *R*_*astro*_ set to 0.1125 mm. The V1 orientation preference map when probed at A: 250, B: 500, C: 750, D: 1000, E: 2500, F: 5000, G: 7500, H: 10000 iterations respectively. I: The stability of the V1 orientation map developed across iterations.

**Fig 7:**
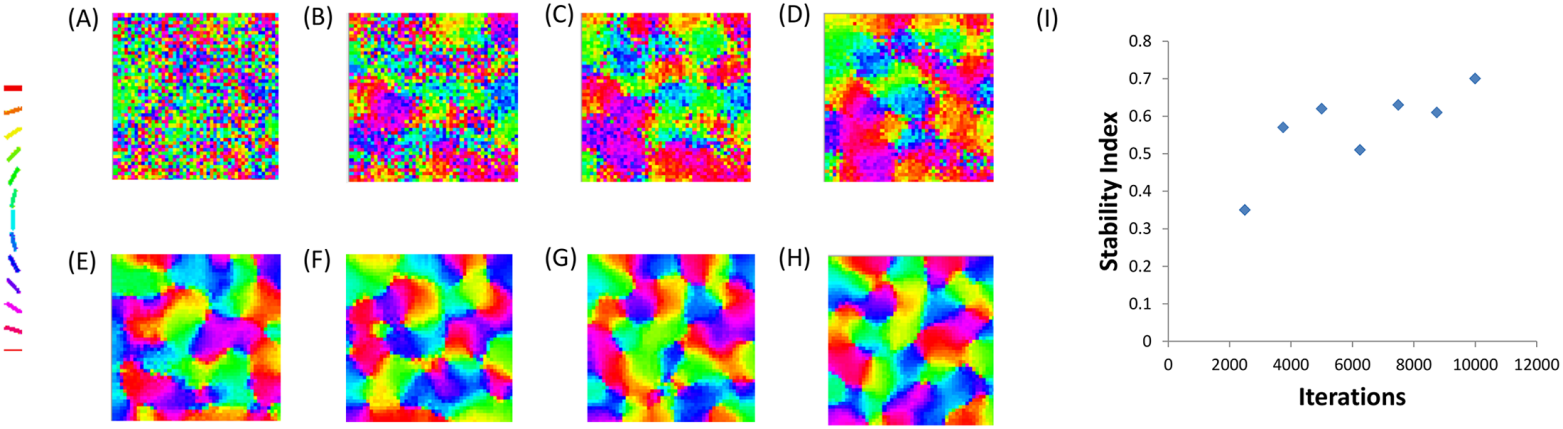
Development of the neuronal (V1) orientation preference map with the astrocyte radius *R*_*astro*_ set to 0.1875 mm. The V1 orientation preference map when probed at A: 250, B: 500, C: 750, D: 1000, E: 2500, F: 5000, G: 7500, H: 10000 iterations respectively. I: The stability of the V1 orientation map developed across iterations.

The development of a few of these maps and their stability indices across iterations are shown in Figs 5, 6, and 7. The V1 orientation preference map is probed at 250, 500, 750, 1000, 2500, 5000, 7500 and 10000 iterations. It is observed that the map developed becomes stable after a few initial iterations, as quantified by the corresponding stability indices. These results demonstrate the model develops stable orientation maps.

**Fig 8:**
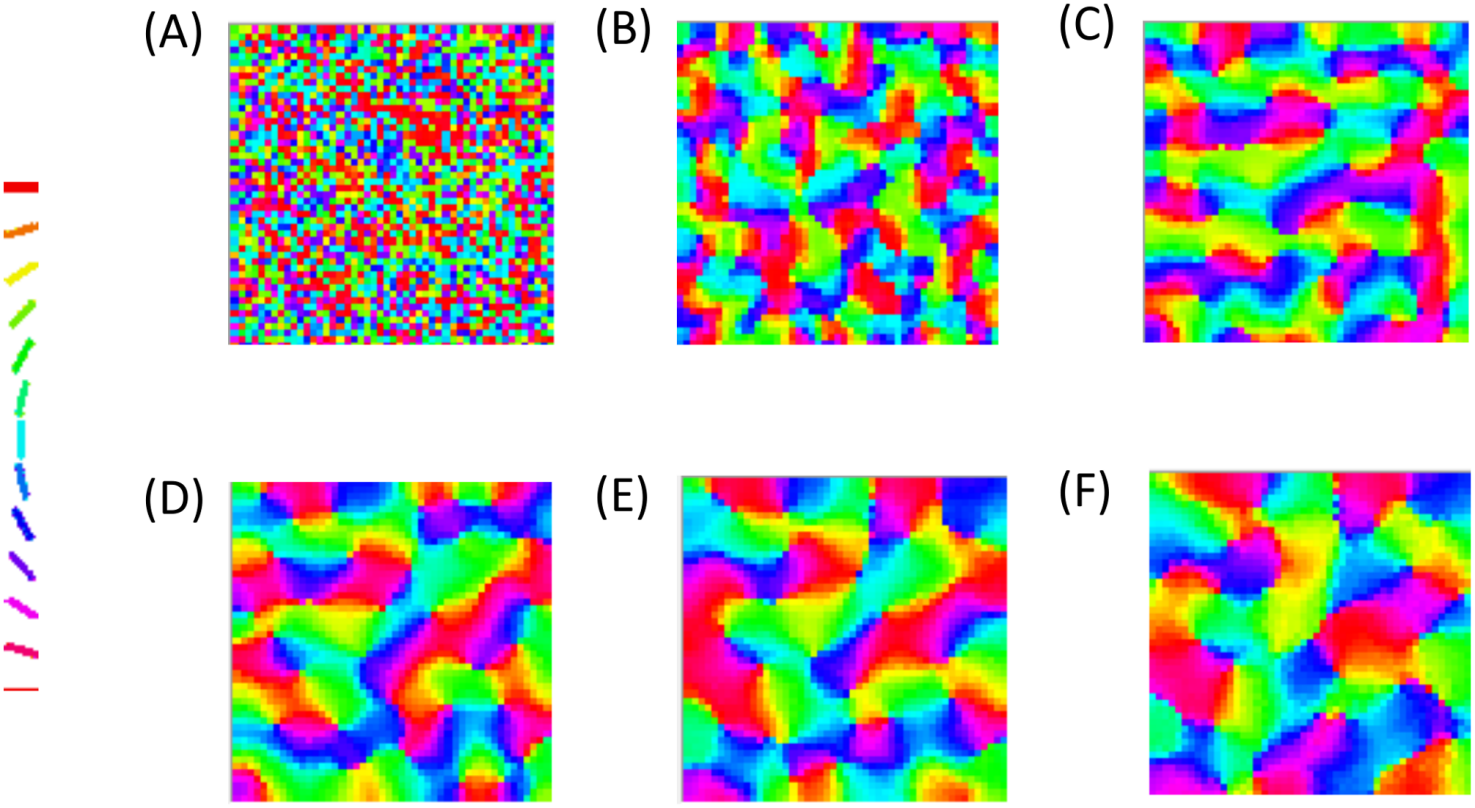
The neuronal (V1) orientation maps developed on varying the radius of astrocytic influence *R*_*astro*_, considering there is no GABA induced gliotransmission. The astrocytic radii used are (A) 0.015 mm, (B) 0.0375 mm, (C) 0.075 mm, (D) 0.1125 mm, (E) 0.15 mm, (F) 0.1875 mm.

**Fig 9:**
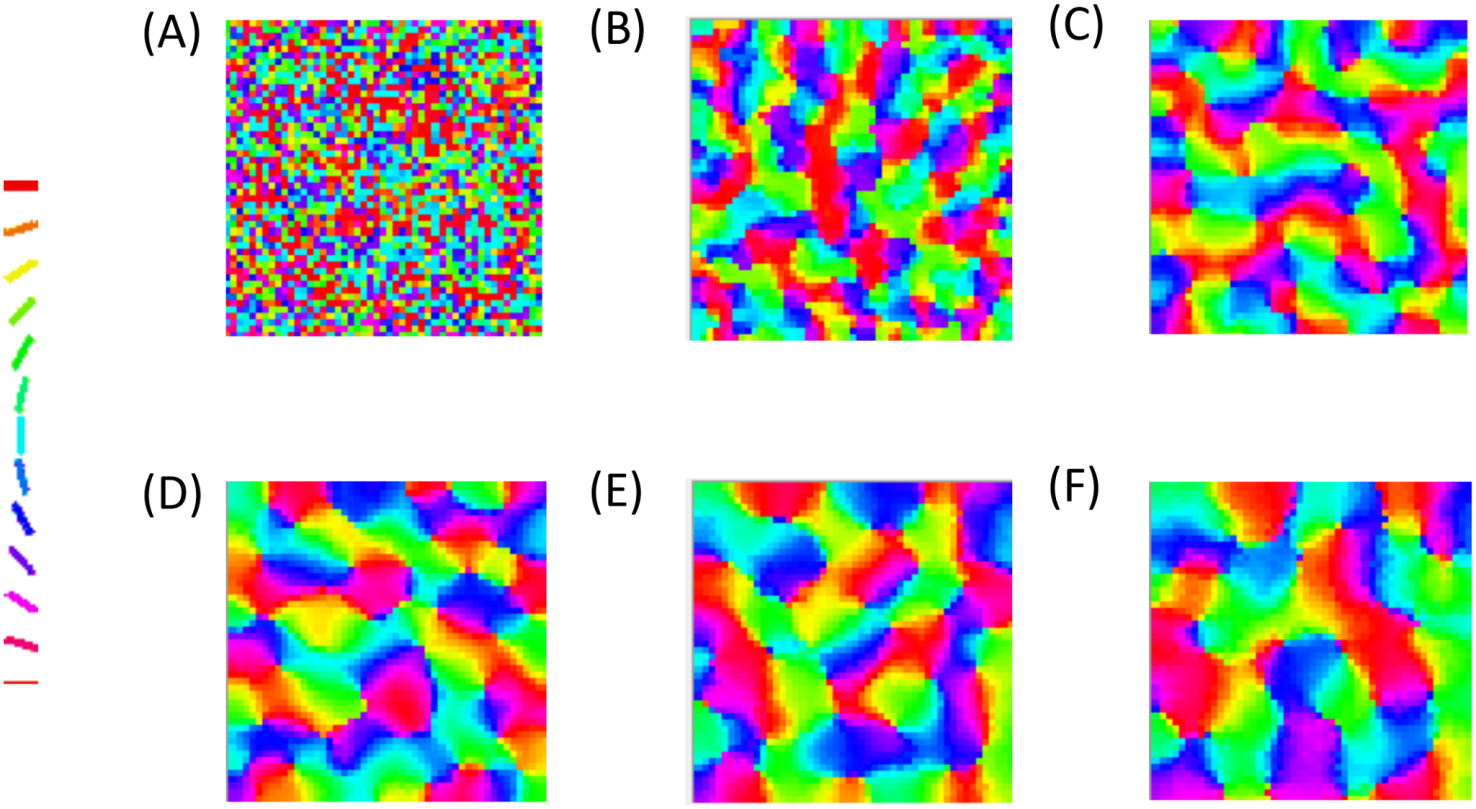
The neuronal (V1) orientation maps developed on varying the radius of astrocytic influence *R*_*astro*_, considering the effect of gap junctions in the astrocyte layer. The astrocytic radii used are (A) 0.015 mm, (B) 0.0375 mm, (C) 0.075 mm, (D) 0.1125 mm, (E) 0.15 mm, (F) 0.1875 mm.

**Fig 10:**
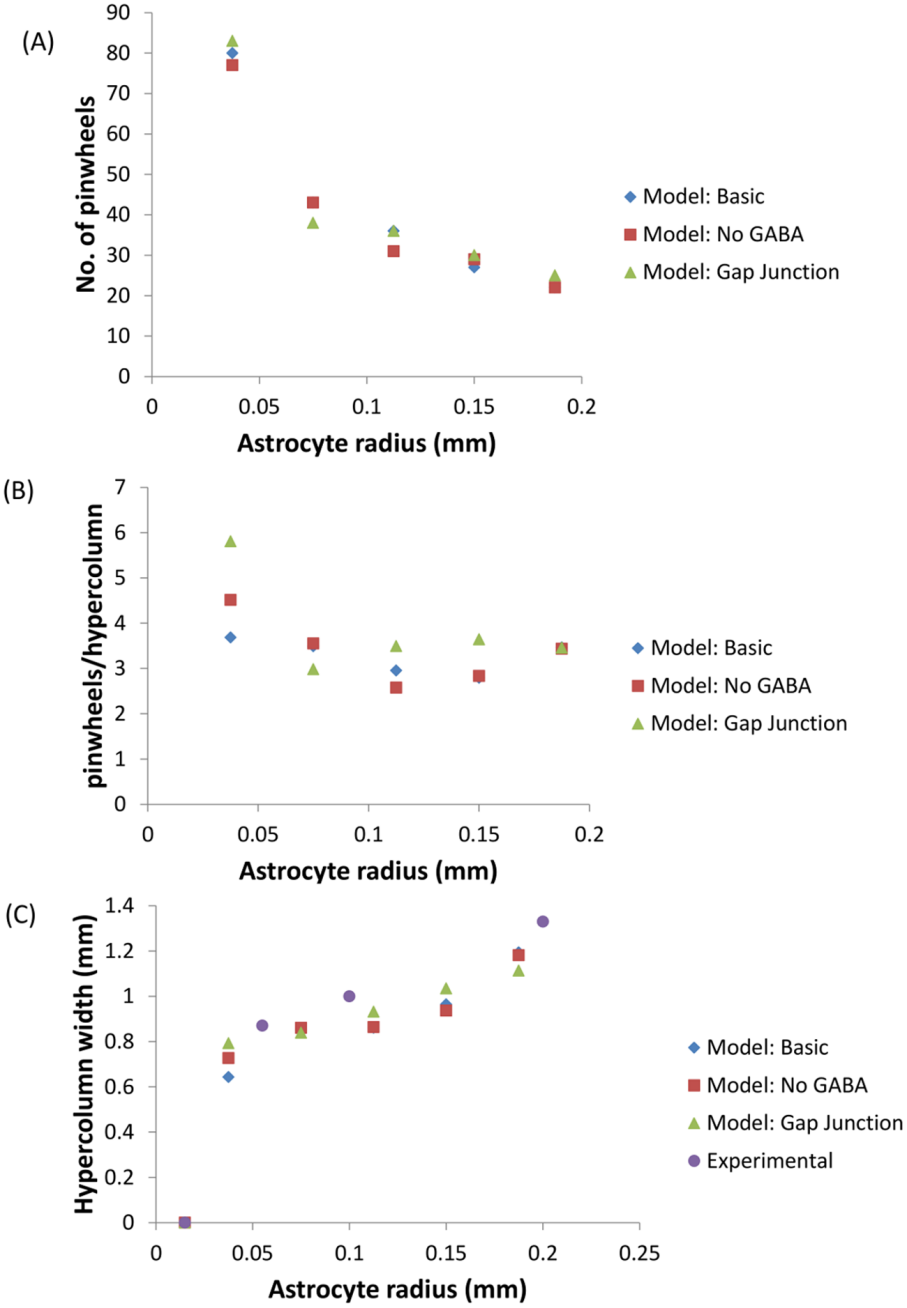
Comparison of features of the developed V1 orientation maps. (A)The number of pinwheels in the simulated region reduces with increasing astrocyte radius; (B) The number of pinwheels per hypercolumn remains approximately constant around *π* for sufficiently large astrocytic radii. When an astrocytic radius of 0.0375 is used the smooth nature of the map begins to disintegrate as evident from the larger number of pinwheels present per hypercolumn; (C) The width of the hypercolumn increases with increasing astrocytic radii.

We also simulate 2 additional conditions which could effect the development of the orientation maps: (1) Considering there is no GABA induced gliotransmission: Since the effect of GABA induced calcium oscillations is not well understood in literature, we also simulate the map development ignoring the corresponding term as described in Eq 9. (2) Considering the effect of gap junctions in the astrocyte layer: The basic simulation does not consider the effect of gap junctions among astrocytes. As described in the methods section, we introduce gap junction by considering excitatory connections among the nearest neighbors in the astrocyte layer. The maps formed for these 2 conditions are shown in Figs 8 and 9 respectively. The maps developed using all 3 conditions (basic, no GABA, Gap junctions) appear visually similar and their features, which are further quantified (See Fig 10), show a similar trend. These results indicate that the correlation between astrocyte radius and hypercolumn widths is robust for all the conditions considered.

The number of pinwheels observed in the neuronal (V1) orientation map in the simulated region (3 x 3 mm) is shown in Fig 10(A). As expected, the number of pinwheels falls with increasing astrocytic radius. The number of pinwheels per hypercolumn remain approximately constant, centered around *π* for the maps in which a clear orientation preference map (OPM) structure is present (Fig 10(B)). However for smaller astrocytic radii the map begins to disintegrate. These results strengthen the hypothesis that the astrocytic radii influence the formation of orientation maps. For higher astrocytic radii the number of pinwheels per hypercolumn stabilizes to values around *π*. This result is in keeping with experimental findings which show that the number of pinwheels per hypercolumn is a constant *π* across species [39]. The trend observed in the simulated widths of the hypercolumn and the corresponding astrocytic radii are comparable with the scant experimental evidence available as shown in Fig 10(C).

The transition from a salt and pepper kind of map to a smooth orientation map could be quantified using 2 methods: (1) Change in the number of pinwheels/ hypercolumn: Experiment results indicate that the number of pinwheels/ hypercolumn remains a constant across species, even with differing hypercolumn widths [39]. Thus, if such a ratio is no longer maintained, the map developed no longer resembles a smooth orientation preference map. However, the map developed is also not truly random since there might be local patches with similar orientation preference. A recent study has shown that in rodents the map only appears to be random, and has significant local orientation similarity [19]. (2) Local similarity in orientation preference: This method quantifies the local smoothness of the map developed. The mean angle of separation between the orientation preference of a node and all others within a predefined radius of interest is computed and compared for different astrocytic radii.

**Fig 11:**
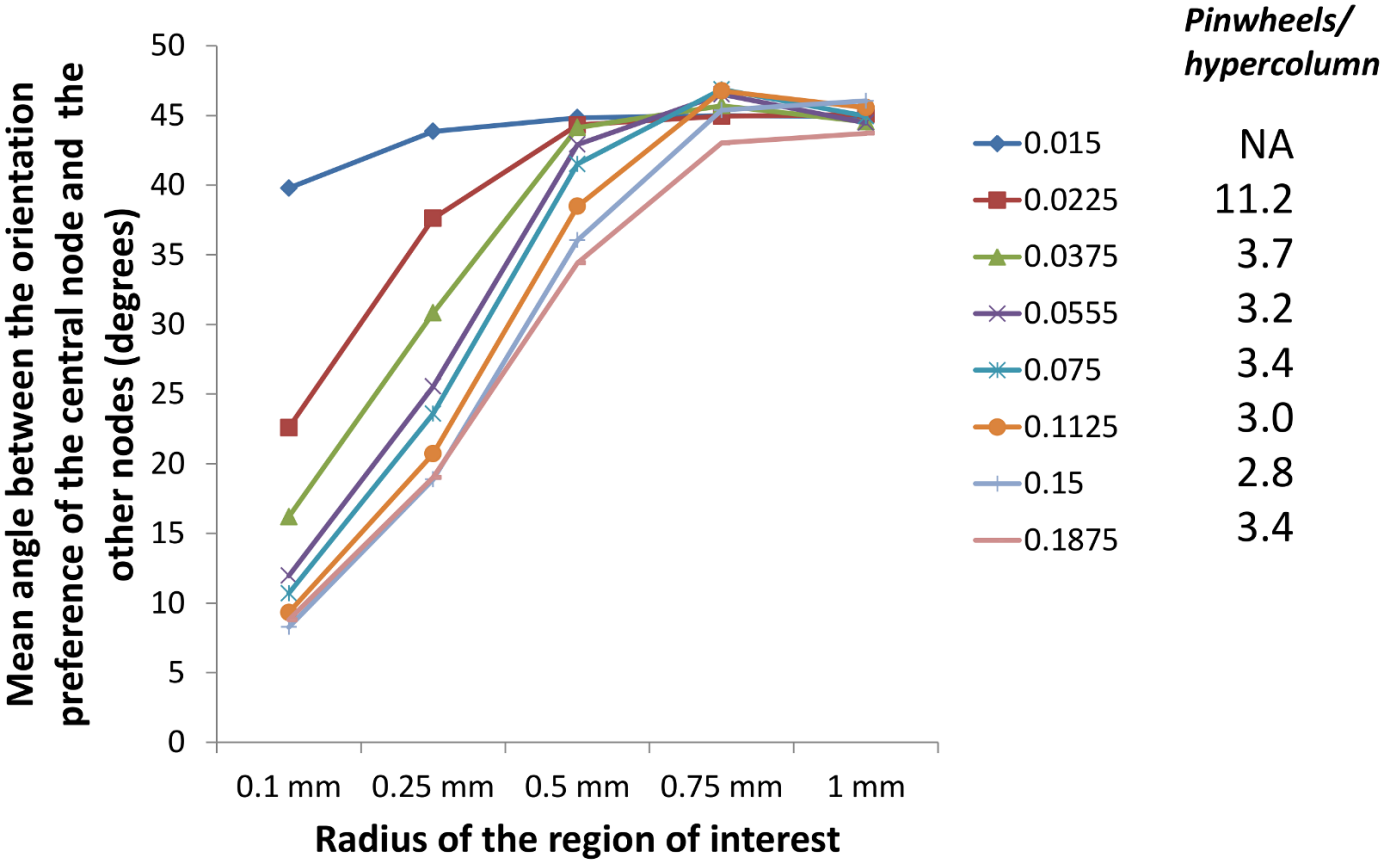
The mean angle of separation between the orientation preference of a node and all others within a predefined radius of interest for different astrocytic radii, compared with the number of pinwheels/hypercolumn in the map developed.

We then compare the results using the 2 methods and observe that a sharp transition between salt and pepper and a smooth orientation maps is absent (Fig 11). Rather, an intermediate state which exhibits local patches of orientation similarity, but lacks the features of a true orientation map is seen.

## Discussion

A fascinating feature of orientation mapping is that not all species display a smooth transition in orientation preferences as we probe along the cortical surface. Rodents, in particular have neuronal columns which are orientation specific but arranged in a seemingly randomized fashion across V1. This kind of organization is referred to as a salt and pepper configuration. The presence or absence of OPMs and their potential consequences for information processing is a topic of current interest.

Another interesting fact in those species which do have OPMs is that the size of the hypercolumn varies from species to species. However the number of pinwheels per hypercolumn appears to remain constant across species. Self organizing mechanisms have been extensively utilized to model OPMs. These models rely on a mechanism that ensures that neighbouring neuronal columns respond to similar features, whereas distant ones to different features. This is invariably implemented by invoking local excitatory and larger inhibitory connections. However cortical inhibitory connections are known to be short range, whereas excitatory ones are longer ranged laterally. These long ranged inhibitory connections have been explained away as long ranged excitatory neurons recruiting local inhibitory neurons, such that the net effect is inhibitory. However, a mechanism that ensures that short range connections are effectively more excitatory than inhibitory has proven elusive. The arguments summarized above have been discussed in detail by Swindale [7]. He postulates the possibility of extracellular diffusion of chemical messengers mediating this short range excitatory connectivity.

However there are a number of issues with the diffusion hypothesis. Firstly diffusion, a passive process, would ensure roughly similar excitatory radii across species and would thus imply by extension similar widths of orientation hypercolumns across species. In reality the widths of hypercolumns vary widely across species. Rodents do not have a smooth topographical variation in orientation preference and hence do not have defined hypercolumns [18]. Thus diffusion alone would not explain the variation in hypercolumn widths.

Secondly the pyramidal apical dendrites, which are used to define the width of a minicolumn (also called microcolumn) are roughly the same (≈ 30*μm*) for the rhesus macaque and the rat [40]. Thus the chemical messenger which diffuses should have similar effects at the level of cortical columns. However as stated earlier this again does not hold true.

Astrocytes are known to regulate both excitatory and inhibitory cortical circuits, via a combination of glutamate and GABA re-uptake by transporters, gliotransmitter release, and regulation of neuronal excitability [27–29, 31, 41, 42]. Indeed, optogenetic astrocyte calcium activation modulates the excitatory-inhibitory balance and increases response selectivity of excitatory neurons within local cortical microcircuits [28]. Thus the extent of astrocyte influence may directly influence the range of local influence in the cortex.

**Fig 12:**
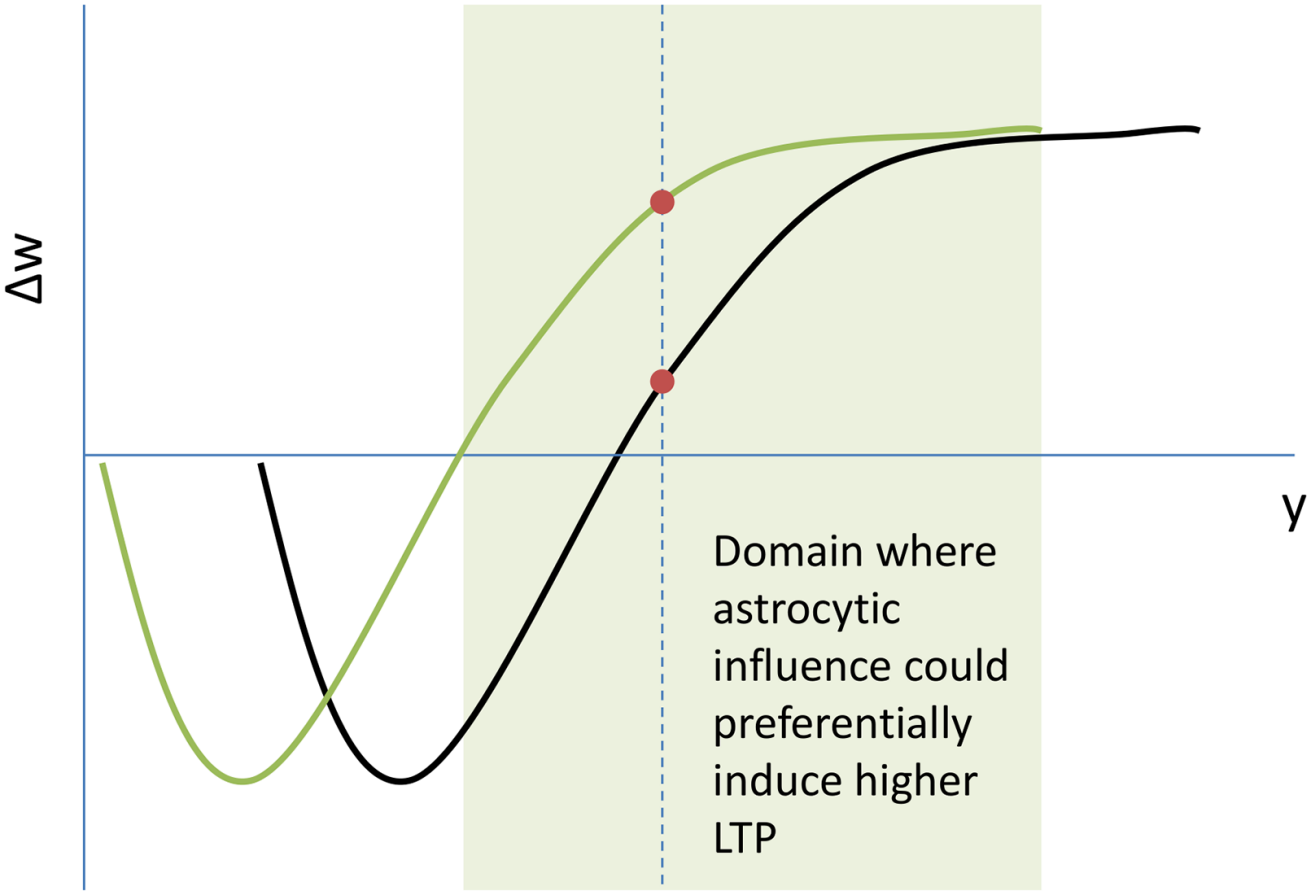
Astrocytic influence on the BCM like curve of synaptic plasticity: Gliotranmitters could induce metaplasticity at the synaptic junction by shifting the BCM like curve to the left. The shaded green regime represents the range of initial weight values which on astrocytic metaplasticity would have higher LTP. The dotted line represents a single instance of the same.

As mentioned earlier, protoplasmic astrocytes are thought to contribute to metaplasticity [30]. As shown in Fig 12, astrocytes release gliotransmitters which are known to shift a BCM like curve to the left, implying greater LTP for lower postsynaptic firing rates in excitatory neurons [31]. This constitutes a greater increase in synaptic strength for those synapses in the vicinity of the gliotransmitter releasing astrocyte.

**Fig 13:**
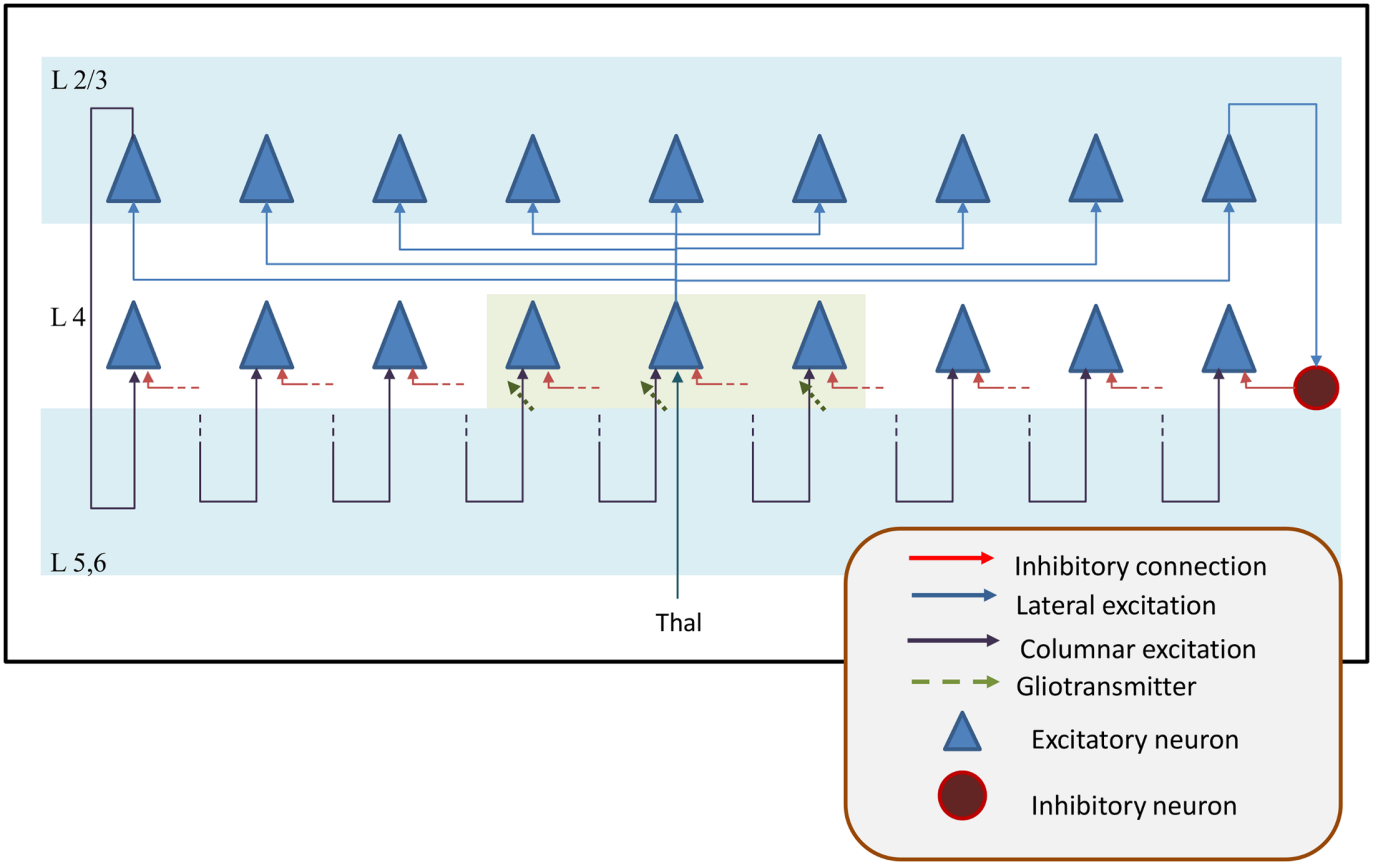
A reduced model of astrocytic influence of a cortical microcircuit.

Now, astrocytes are understood to release gliotransmitters in correlation with their internal calcium levels [30]. Glutamate in the synaptic cleft, either via receptors or transporters, mediates the calcium levels in the engulfing astrocyte. Astrocytes associated with synapses corresponding to those layer 4 pyramidal neurons, which receive direct thalamic input, would have greater calcium levels as compared to other astrocytes. Hence the metaplasticity induced in the neighboring excitatory synapses in the domain of these astrocytes would also be more pronounced. Over time, this would lead to a greater excitatory drive for those neurons in the vicinity of the neuron receiving the direct thalamic input, as shown in Fig 13.

We hypothesize that astrocytes influence local synapses and specify the radius of lateral excitatory connections. This in turn influences the size of hypercolumns across species. A comparative table specifying hypercolumn widths and astrocyte radii is specified in Table 2. In the standard GCAL model there is a constraint placed on the maximum radius of the lateral excitatory connections [35]. Indeed, this constraint is necessary to ensure that the lateral excitatory connections are shorter in range than the lateral inhibitory ones. Such a configuration is essential for the development of the self organized orientation map. In our proposed model the lateral excitatory connections have no defined maximum radius. The limit on the lateral excitatory connections is implicitly imposed due to the fact that the astrocytes control the BCM threshold of the excitatory neuronal synapses within their (astrocyctic) radius of influence and ensures LTP. For synapses outside the astrocytic radius, the threshold is such that LTD occurs and these connections are pruned off automatically.

**Table 2:** Comparison of astrocytic radii and hypercolumn widths.

Species	Hypercolumn width	Astrocyte radius
Rodent	NA	28 *μm* [45]
Cat	1 *mm* [39]	100 *μm* [46]
Human	1.33 *mm*	200 *μm* [37]
Tree Shrew	0.62 *mm* [39]	?
Galago	0.68 *mm* [39]	?
Ferret	0.87 *mm* [39]	55 *μm* [47]

As a computational principle, any mechanism that can control the lateral excitatory radius with respect to the lateral inhibitory radius, could produce similar maps as those shown in this manuscript. However, several lines of evidence indicate that astrocytes are strongly involved in this mechanism. First, astrocytes have been shown to regulate the excitatory to inhibitory balance in local neuronal circuits [28]. Importantly, astrocytes express transporters for both glutamate and GABA, and can thus regulate the strength of both excitatory and inhibitory synaptic transmission. Second, they have a significant role in regulating local synaptic plasticity, in particular local neuronal excitation, via a range of mechanisms that include modulation of NMDA receptors as well as integrating other plasticity-mediating neuromodulators such as acetylcholine, noradrenaline [43, 44]. Together, these effects are well placed to implement the BCM rule. Third, astrocytes are known to form microdomains with less than 10% overlap [37]. Thus, the astrocytic organization automatically results in the formation of local domains, which are influenced by these transmitters/modulators. Fourth, the radii of these local domains are known to be less than the effective lateral inhibitory radius, thus resulting in the required short range excitation and longer range inhibition.

We simulate the development of orientation maps with varying hypercolumn widths, by simply varying the radius of astrocytic connections using the LISSOM model with an additional layer simulating the astrocytic activation. We observe that increasing the astrocytic radius, and thereby the effective radius of lateral excitatory connections in the V1 neuronal layer, the width of the hypercolumn developed shows a proportionate increase. When the effective lateral excitatory radius is reduced so as to almost prevent any similarity in orientation preference of neighboring neurons, the OPM disappears and a salt and pepper configuration of neuronal arrangement of orientation preference is seen.
